# DEFYING THE ODDS: EXCEPTIONAL HOME HEALTH AGENCIES SERVING HIGH PROPORTIONS OF BLACK PATIENTS

**DOI:** 10.1093/geroni/igad104.0386

**Published:** 2023-12-21

**Authors:** Shekinah Fashaw-Walters

**Affiliations:** University of Minnesota, Minneapolis, Minnesota, United States

## Abstract

Healthcare providers with the highest proportions of Black patients are typically assumed to be lower quality, whether they be hospitals, nursing homes, or primary care physicians. However, little research examines the providers that “defy these odds” and rise to the top of the quality ratings. Each year, Medicare home health provides skilled nursing and therapy services to over 3 million homebound older adults with physical and cognitive impairments, and compared to the general Medicare population, Medicare home health patients are more likely to identify as Black. This mixed-methods (QUANT-->QUAL) project uses an assets-based approach as well as the tenets of the Public Health Critical Race Praxis to understand home health providers that are generally assumed to be low-quality providers – that is those who have the highest proportions of Black patients – but instead are high quality providers. This study uses star ratings and the Post-Acute Care Public Use File for home health agencies to characterize and identify agencies with high quality care and a high proportion of Black patients. Publicly available data allows us to characterize the structures and outcomes of these unique home health agencies; while primary data collection (via case studies of exceptional agencies) will allow us to report on their unique processes of care. Data collection and analyses are currently underway and will be completed by May 2023. In 2019, 28% of home health agencies were identified as having high-quality with a high proportion of Black patients, we have selected three agencies for case studies.

